# P2X_2_ receptors supply extracellular choline as a substrate for acetylcholine synthesis

**DOI:** 10.1002/2211-5463.13332

**Published:** 2021-11-27

**Authors:** Takuma Maruyama, Asuka Mano, Toshiyuki Ishii, Yoshihiko Kakinuma, Makoto Kaneda

**Affiliations:** ^1^ Department of Physiology Nippon Medical School Tokyo Japan

**Keywords:** acetylcholine, choline, choline transporter, cholinergic neuron, HEK293T cell, P2X_2_ receptor

## Abstract

Acetylcholine (ACh), an excitatory neurotransmitter, is biosynthesized from choline in cholinergic neurons. Import from the extracellular space to the intracellular environment through the high‐affinity choline transporter is currently regarded to be the only source of choline for ACh synthesis. We recently demonstrated that the P2X_2_ receptor, through which large cations permeate, functions as an alternative pathway for choline transport in the mouse retina. In the present study, we investigated whether choline entering cells through P2X_2_ receptors is used for ACh synthesis using a recombinant system. When P2X_2_ receptors expressed on HEK293 cell lines were stimulated with ATP, intracellular ACh concentrations increased. These results suggest that P2X_2_ receptors function in a novel pathway that supplies choline for ACh synthesis.

## Graphical abstract

Acetylcholine (ACh) is synthesized from choline. In cholinergic neurons, choline uptake through high‐affinity choline transporters was considered to be the only pathway for ACh synthesis. In the present study, we report that P2X_2_ receptors function in a novel pathway of choline transport for ACh synthesis.

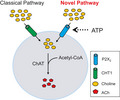

**Abbreviations**
Ach: acetylcholine
ChAT: choline acetyltransferase
CHT1: high‐affinity choline transporter
CTL: choline transporter‐like protein
HEK293 cells: human embryonic kidney 293‐derived cells
HEK293T cells: human embryonic kidney 293‐derived cells that express the simian virus 40 T antigen
PB: phosphate buffer

Acetylcholine (ACh) synthesized from choline in cholinergic neurons [1, 2, 3, 4] is a major excitatory neurotransmitter that plays an important role in both the central and peripheral nervous systems [5, 6, 7, 8, 9, 10]. Choline uptake by cholinergic neurons is mediated by three types of choline transporters [11, 12], the distribution of which differs. The high‐affinity choline transporter (CHT1) is exclusive to the axon terminals of cholinergic neurons [11, 13, 14, 15, 16], while the intermediate‐affinity transporter, choline transporter‐like protein 1 (CTL1), or members of low‐affinity choline transporters, polyspecific organic cation transporters (OCT1–3) and CTL2, are ubiquitous throughout the cell surface of multiple tissues [11, 12]. The functions of these transporters also differ. A previous study demonstrated that ACh concentrations were reduced in the cerebral cortex, hippocampus, and striatum of CHT1 +/− mice [17]. The application of hemicholinium‐3 (HC‐3), an inhibitor of CHT1 and CTL1, suppressed the release of ACh in the rat striatum [18]. Furthermore, reductions in the synthesis of ACh following the application of HC‐3 were reported in the rat retina [19]. Choline taken up through CHT1 is used in the synthesis of ACh (Fig. 3A) [11, 20, 21], whereas that taken up by intermediate‐ or low‐affinity choline transporters is used to synthesize phospholipids [11, 12, 22]. Therefore, the activity of cholinergic neurons has been assessed based on the activity of CHT1 [23].

In the retina, ACh is released from cholinergic amacrine cells [19, 24]. We recently demonstrated that P2X_2_ receptors, a subtype of P2X purinoceptors that form nonselective cation channels [25, 26], function as an alternative pathway for choline entry in the cholinergic amacrine cells of the mouse retina [27]. However, it currently remains unclear whether choline that enters through P2X_2_ receptors is used to synthesize ACh because of the difficulties associated with measuring ACh concentrations in the cholinergic amacrine cells of the mouse retina.

Therefore, we herein investigated whether P2X_2_ receptor‐expressing human embryonic kidney 293‐derived cells, which express the simian virus 40 T antigen (HEK293T cells), synthesize ACh from choline entering through P2X_2_ receptors because human embryonic kidney 293‐derived cells (HEK293 cells) synthesize ACh [28] (Fig. 3B). The results obtained revealed a significant increase in intracellular ACh concentrations ([ACh]_i_) in P2X_2_ receptor‐expressing HEK293T cells when P2X_2_ receptor‐mediated choline influx was activated. These results support choline transport through P2X_2_ receptors functioning as a second pathway for ACh synthesis in cholinergic neurons.

## Materials and methods

### Construction of an expression vector

A cDNA fragment of the P2X_2_ receptor was amplified from a mouse *P2rx2*/pcDNA3 vector by PCR using the following primers (5′‐CCGAGAATTCGCCGCCATGGCCGCTG‐3′ and 5′‐CCGAGTCGACTATCAAAGTTGGGCCAAACCTT‐3′); cDNA was inserted into the EcoRI/SalI site of a pIRES2‐AcGFP1‐Nuc vector (TaKaRa Bio, Shiga, Japan). Since nuclear localization signals are fused to the GFP coding region, GFP is expressed in the nucleus.

### Cell culture and transfection

HEK293T cells (RIKEN Bio‐Resource Center, Tsukuba, Japan) were cultured in HEK medium consisting of Dulbecco's modified Eagle's medium (DMEM; Sigma‐Aldrich, Taufkirchen, Germany) containing 10% FBS (Biowest, Nuaillé, France), penicillin (100 U·mL^−1^), and streptomycin (100 µg·mL^−1^; P/S; Nacalai Tesque Inc., Kyoto, Japan). HEK293 cells stably expressing murine choline acetyltransferase (ChAT; ChAT‐HEK293 cells) [29] were cultured in ChAT‐HEK medium consisting of DMEM containing 10% FBS and hygromycin B (500 µg·mL^−1^; Nacalai Tesque, Inc., Kyoto, Japan). HEK293T or ChAT‐HEK293 cells were plated on 10‐cm dishes (2 × 10^6^ cells per dish) maintained at 37 °C in a 5% CO_2_/air atmosphere. HEK293T or ChAT‐HEK293 cells were transfected with the pP2X_2_‐IRES2‐AcGFP1‐Nuc vector (10 μg plasmid DNA per dish) using Lipofectamine 3000 (Invitrogen, Carlsbad, CA, USA) according to the manufacturer's instructions, and transfected cells were incubated for 2 days.

### Immunocytochemistry

Immunocytochemistry was performed as described in our previous studies [27, 30]. In brief, HEK293T or ChAT‐HEK293 cells were fixed with 4% paraformaldehyde (w/v) in 0.1 m phosphate buffer (PB) for 30 min, permeabilized for 15 min in 0.1 m PB containing 0.3% Triton‐X 100, and blocked with 0.1 m PB containing 1% Block Ace (Dainippon Seiyaku, Osaka, Japan) at room temperature for 60 min. Samples were reacted with a rabbit anti‐P2X_2_ receptor (working dilution 1 : 500, Invitrogen) in 0.1 m PB containing 0.4% Block Ace at 4 °C for 24 h. Samples were then allowed to react with Alexa 594‐conjugated goat anti‐rabbit IgG (working dilution 1 : 500, Invitrogen) in 0.1 m PB at room temperature for 2 h and then counterstained with 4′, 6‐diamidino‐2‐phenylindole (DAPI; working dilution 1 : 500, Nacalai Tesque, Inc., Kyoto, Japan). Fluorescent images were observed under a confocal microscope (FV1200; Olympus, Tokyo, Japan) at an excitation wavelength of 488 nm for GFP (bandpass 505–540 nm), 559 nm for Alexa 594 (bandpass 575–675 nm), and 405 nm for DAPI (bandpass 430–470 nm).

### HPLC

HEK293T or ChAT‐HEK293 cells were incubated in one of the choline‐containing extracellular solutions shown in Table 1 with or without 30 μm ATP for 5 min. After washing with Dulbecco's phosphate‐buffered saline [free of Ca and Mg; D‐PBS (−)], cells were cultured in HEK or ChAT‐HEK medium for 25 min to allow for ACh synthesis. After washing with D‐PBS (−) twice, collected cells were treated with 500 μL PCA solution containing 0.1 m perchloric acid, 0.1 mm EDTA, 100 μm isopropyl homocholine, and 0.1 mm eserine, a blocker of cholinesterase. Samples were centrifuged at 20,000 ***g*** at 0 °C for 15 min. After adjusting pH to approximately 7.0 using 1 m KHCO_3_, the supernatant was collected and filtered (Millipore Ultrafree MC 10,000 MW cutoff UFC3LGC00; Millipore, Billerica, MA, USA) by centrifugation (14,000 ***g*** at room temperature for 20 min). The sample (10 μL) was injected into the HPLC system (HTEC‐500; Eicom, Kyoto, Japan) equipped with a guard column (CH‐Gel), separation column (AC‐Gel), and immobilized enzyme column (AC‐Enzyme Pack II) in series. The composition of the mobile phase was 50 mm KHCO_3_, 300 mg·L^−1^ sodium 1‐decanesulfonate, and 50 mg·L^−1^ EDTA. The flow rate of the mobile phase was 0.15 mL·min^−1^. Peak data were recorded and analyzed on a computer. In our system, the detection limit of the intracellular choline concentration was 0.1 nm. The choline concentration in each sample was estimated using the internal standard, isopropyl homocholine.

**Table 1:** Composition of extracellular solutions (in mm).

	NaCl	Choline Cl	KCl	MgCl_2_	CaCl_2_	HEPES	Glucose	Sucrose
Choline‐rich	0	135	5	1	2	5	10	0
Choline‐Na	134.9	0.1	5	1	2	5	10	0
Choline	0	0.1	5	1	2	5	10	234

### Western blot analysis

HEK293T or ChAT‐HEK293 cells were washed twice with D‐PBS (−) and directly lysed with 1 × SDS/PAGE sample buffer (62.5 mm Tris/HCl, 3 w/v% SDS, 7.5% glycerol, 0.005 w/v% bromophenol blue, and 50 mm dithiothreitol). All samples were run on e‐PAGEL (ATTO, Tokyo, Japan) and then transferred onto a polyvinylidene fluoride membrane (Millipore). After blocking with 5% skim milk in Tris‐buffered saline containing 0.1% Tween‐20, membranes were processed through sequential incubations with goat anti‐ChAT (working dilution 1 : 1000; Chemicon, Temecula, CA, USA) overnight and then with a horseradish peroxidase‐conjugated anti‐goat IgG antibody (working dilution 1 : 10,000, Jackson Laboratory, Bar Harbor, ME, USA) for 2 h. An internal standard, β‐actin, was detected using anti‐β‐actin antibodies followed by a horseradish peroxidase‐conjugated anti‐mouse IgG antibody (working dilution 1 : 3000, Cell Signaling Technology, Danvers, MA, USA). Bound antibodies were visualized using a chemiluminescence imager (Amersham, Little Chalfont, UK), detected with Light‐Capture (AE‐6972; ATTO), and quantified using CS Analyzer (ver. 3.00 software; ATTO).

### Statistical analysis

**Fig. 1:**
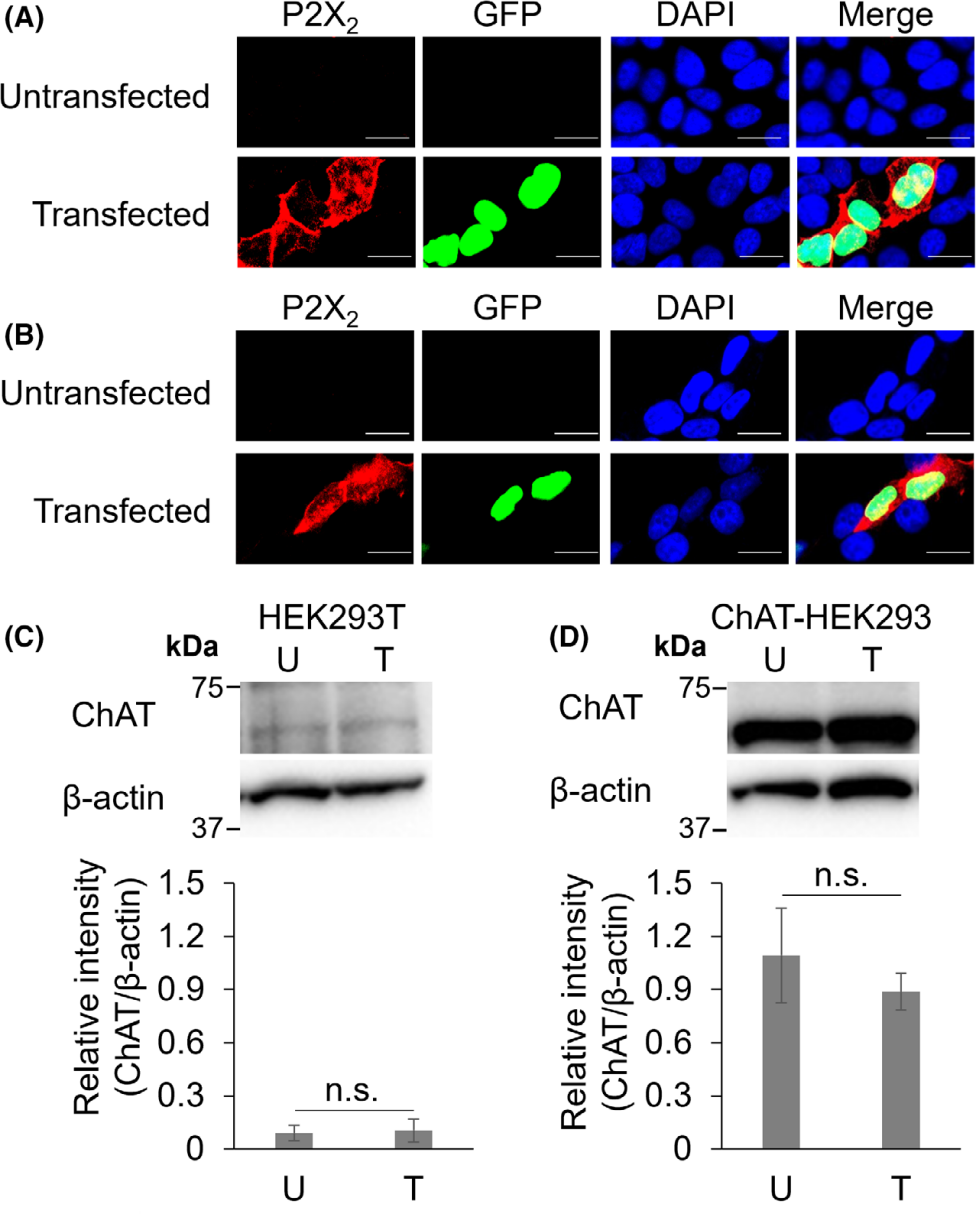
Expression of the P2X_2_ receptor and ChAT in HEK293T and ChAT‐HEK293 cells. (A and B) Photomicrographs showing the immunocytochemical distribution of P2X_2_ receptors and GFP in HEK293T cells (A) and ChAT‐HEK293 cells (B). Upper panels show the results of untransfected cells, and lower panels show those of cells transfected with the pP2X_2_‐IRES2‐AcGFP1‐Nuc vector. Scale bars; 20 μm. (C, D) left, western blots of ChAT and β‐actin in HEK293T cells (C) and ChAT‐HEK293 cells (D). Right, relative intensity of ChAT. The intensities of bands in ChAT were measured and normalized by the values of β‐actin. U: Samples of untransfected cells. T: Samples of cells transfected with the pP2X_2_‐IRES2‐AcGFP1‐Nuc vector. The number of samples was 6 for all experimental conditions. Bars are shown as the mean ± SD; n.s., not significant (Student's *t*‐test).

**Fig. 2:**
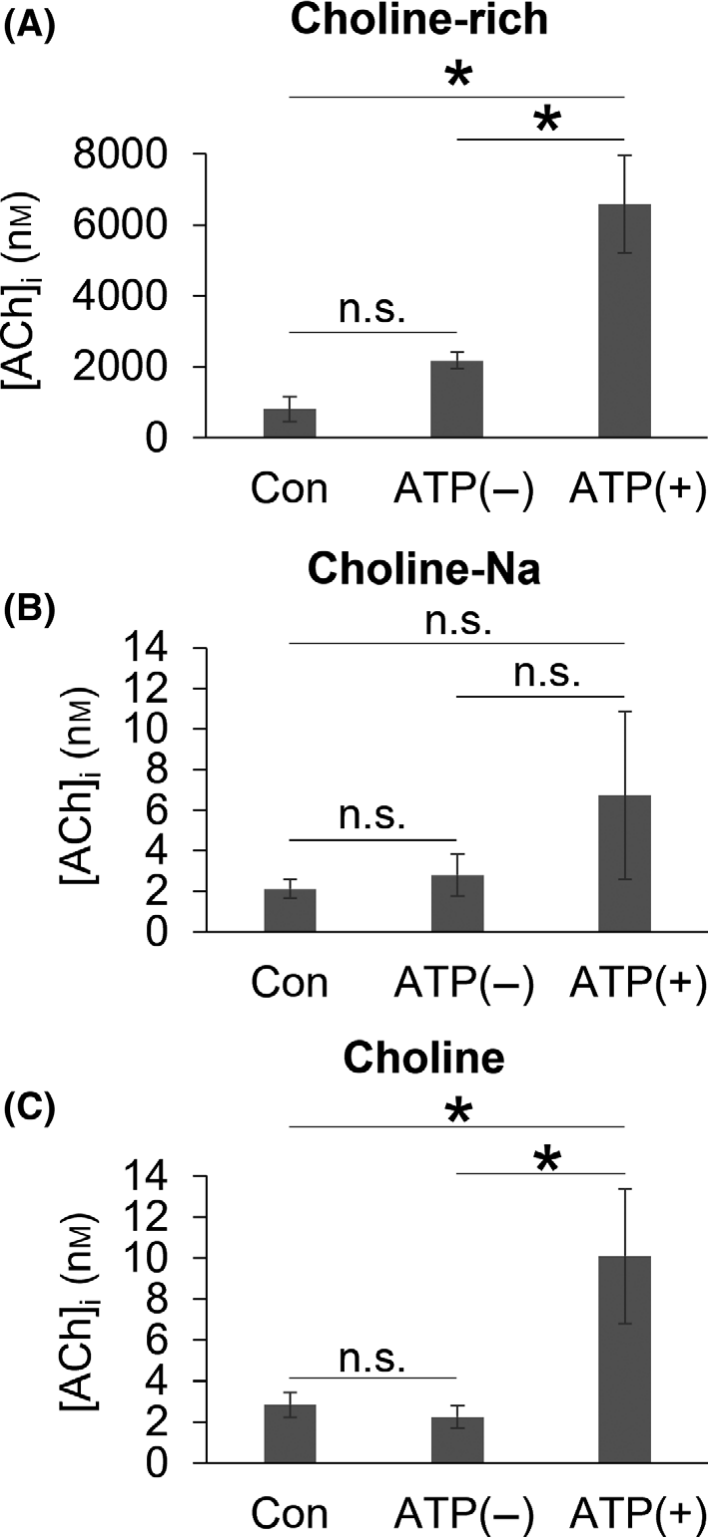
ACh synthesis in transfected HEK293T cells and ChAT‐HEK293 cells expressing the fusion protein of P2X_2_ receptors and GFP. (A) The intracellular concentration of ACh ([ACh]_i_) in ChAT‐HEK293 cells in choline‐rich solution (Table 1). Sample numbers were 4 (Con), 4 (ATP (−)), and 5 (ATP (+)). (B) [ACh]_i_ in HEK293T cells in choline‐Na solution (Table 1). Sample numbers were 7 (Con), 7 (ATP (−)), and 7 (ATP (+)). (C) [ACh]_i_ in HEK293T cells in choline solution (Table 1). Sample numbers were 9 (Con), 9 (ATP (−)), and 13 (ATP (+)). Con, untransfected cells incubated in culture medium (HEK medium for HEK293T cells or ChAT‐HEK medium for ChAT‐HEK293 cells) for 5 min without the application of ATP. ATP (−), transfected cells incubated in one of the choline‐containing solutions shown in Table 1 for 5 min without 30 µm ATP. ATP (+), transfected cells incubated in one of the choline‐containing solutions shown in Table 1 for 5 min with 30 µm ATP. Bars are shown as the mean ± SE; n.s., not significant; *P < 0.05 (the Steel‐Dwass test).

The significance of differences was calculated with Student's *t*‐test (Fig. 1C,D) or Steel‐Dwass test (Fig. 2A–C). Details on the results of individual statistical analyses are described in the figure legends.

## Results

### The overexpression of the P2X_2_ receptor does not affect ChAT expression levels

We examined whether the overexpression of the P2X_2_ receptor in HEK293T or ChAT‐HEK293 cells affected ChAT expression levels. Immunoreactivity for P2X_2_ receptors was detected in GFP‐positive cells in both cell lines, but not in untransfected cells (Fig. 1A,B). We then investigated whether untransfected HEK293T cells expressed ChAT similar to HEK293 cells [28]. The results obtained showed that ChAT was also expressed in HEK293T cells (Fig. 1C). Under untransfected conditions, ChAT expression levels were higher in ChAT‐HEK293 cells than in HEK293T cells (Fig. 1C,D). The transfection of the pP2X_2_‐IRES2‐AcGFP1‐Nuc vector did not affect ChAT expression levels in either cell line (Fig. 1C,D).

### Choline that enters cells through P2X_2_ receptors is synthesized into ACh

In HEK293T cells expressing P2X_2_ receptors, we previously reported that the application of ATP opened P2X_2_ receptors, resulting in the extracellular influx of choline into the intracellular space [27]. In the present study, to clarify whether choline that enters through the P2X_2_ receptor is used for ACh synthesis, we applied ATP to ChAT‐HEK293 cells in choline‐rich solution to maximize choline influx and the opportunity for ACh synthesis (Table 1). As a control, we incubated ChAT‐HEK293 cells in culture medium (ChAT‐HEK medium) for 5 min to assess basal [ACh]_i_ (Fig. 2A, Con). In P2X_2_ receptor‐expressing ChAT‐HEK293 cells, the application of ATP significantly increased [ACh]_i_ (Fig. 2A, ATP (+)). A simple incubation of P2X_2_ receptor‐expressing ChAT‐HEK293 cells in choline‐rich solution did not significantly increase [ACh]_i_ (Fig. 2A, ATP (−)).

We then investigated whether choline entering through P2X_2_ receptors induced significant increases in [ACh]_i_ under physiological conditions. We activated choline influx through P2X_2_ receptors in a physiologically relevant concentration of choline and measured [ACh]_i_. Since there are currently no available data on choline concentrations at the synaptic cleft, we estimated its concentration at this location. Based on the concentration of choline in plasma (5–10 μm) [11, 23] and the maximum concentration of ACh at the synaptic cleft (>1000 μm) [31], we assumed that the concentration of choline at the synaptic cleft was 100 μm. In subsequent experiments, we used P2X_2_ receptor‐expressing HEK293T cells to efficiently separate the peak of ACh from that of choline in HPLC. We speculated that the synaptic cleft is filled with extracellular fluid; therefore, we investigated whether the influx of choline from Na‐containing solution (Table 1, choline‐Na solution) induced a significant increase in [ACh]_i_. However, the application of ATP did not induce a significant increase in [ACh]_i_ (Fig. 2B). We then replaced extracellular cations with equimolar sucrose and applied ATP to clarify the role of the P2X_2_ receptor in ACh synthesis through the influx of choline because sucrose does not compete with choline^+^ at the pores of P2X_2_ receptors (Table 1, choline solution). In choline solution, the application of ATP significantly increased [ACh]_i_ (Fig. 2C).

## Discussion

**Fig. 3:**
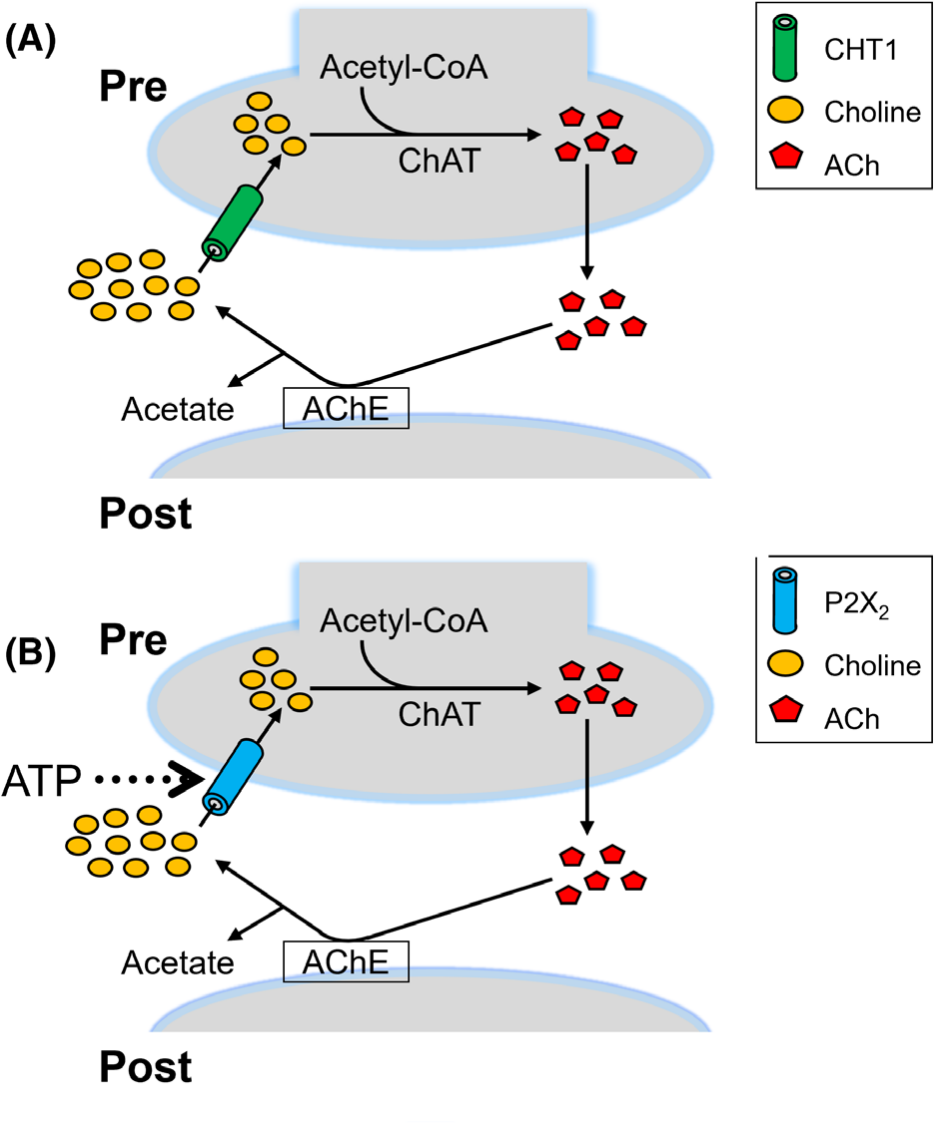
Classical and novel pathways for ACh synthesis and the turnover of ACh in cholinergic neurons. (A) Classical pathway. The high‐affinity choline transporter (CHT1) located on the presynaptic terminal takes up choline. ChAT synthesizes ACh from choline and acetyl CoA. ACh is released into the extracellular space as a neurotransmitter. Released ACh is degraded into choline and acetate by cholinesterase (ChE) at the synaptic cleft. Choline is recycled by CHT1. (B) Novel pathway. The opening of cation channels coupled with P2X_2_ receptors located on presynaptic terminals induces the influx of choline. Choline is used as a substrate for ACh synthesis. The turnover of synthesized ACh follows the same scheme as that shown in the classical pathway.

In cholinergic neurons, choline uptake through CHT1 is regarded as the only source for ACh synthesis (Fig. 3A). In the present study, we showed that choline entering through P2X_2_ receptors is also used for ACh synthesis. Our results support the hypothesis that P2X_2_ receptors function as a novel choline transport pathway for ACh synthesis (Fig. 3B).

The present results revealed an increase in [ACh]_i_ in P2X_2_ receptor‐expressing ChAT‐HEK293 cells in choline‐rich solution when cells were stimulated with ATP. Since intermediate‐ or low‐affinity choline transporters are expressed and continuously function in various tissues [11, 12], we speculated that the increase observed in [ACh]_i_ was induced by the greater uptake of choline through these choline transporters. However, the significant increase observed in [ACh]_i_ cannot be explained by choline uptake through these choline transporters because their activity remains stable in the presence and absence of ATP. However, the slight increase detected in [ACh]_i_ after the incubation in choline‐rich solution without ATP (Fig. 2A, ATP (−)) may be explained by choline uptake through these choline transporters.

We also demonstrated a significant increase in [ACh]_i_ when the extracellular concentration of choline was 100 μm in choline solution. However, we did not detect a significant increase in [ACh]_i_ when the extracellular concentration of choline was 100 μm in choline‐Na solution even though an increase in [ACh]_i_ appeared to occur (Fig. 2B). There are three possible explanations for this difference. It may be explained by competition between Na^+^ and choline^+^ at the cation channels of P2X_2_ receptors, similar to the anomalous mole fraction effects reported in Ca channels [32, 33, 34, 35, 36]. The permeability of choline^+^ to the P2X_2_ receptor is less than that of Na^+^ [27]. We observed a significant increase in [ACh]_i_ in choline solution because choline^+^ freely permeates the cation channel of P2X_2_ receptors without competition from Na^+^ (Fig. 2A,C). Furthermore, the P2X_2_ receptor‐coupled increase in [ACh]_i_ synthesis may be masked by the endogenous synthesis of ACh in HEK293T cells. Previous studies reported the low endogenous expression of the P2X_2_ receptor in HEK293T cells [37]. If the extracellular level of ATP is high due to a leak from damaged cells [25, 38] or release induced by a mechanical stimulation [39, 40], ATP stimulates choline influx through the endogenous P2X_2_ receptor, thereby increasing the background level of [ACh]_i_. In addition, although we estimated that the concentration of choline at the synaptic cleft was 100 μm, its actual concentration may be >100 μm because ACh concentrations at the synaptic cleft may increase to >1 mm [31]. If ACh is immediately degraded by cholinesterase under these conditions, the concentration of choline at the synaptic cleft may become higher than our estimate. There is currently no available data on the concentrations of various cations at the synaptic cleft or the distribution of P2X_2_ receptors at synapses; therefore, further studies are warranted.

We previously reported that choline entered the intracellular space through P2X_2_ receptors as ionic currents in the cholinergic neurons of the retina [27]. Choline transport by P2X_2_ receptors is only active when P2X_2_ receptors are stimulated, and the speed of choline transport by P2X_2_ receptors may be assessed based on the concentration gradient between intra‐ and extracellular choline concentrations. These characteristics differ from conventional choline transport by CHT1 in cholinergic neurons. We also previously demonstrated that two types of retinal cholinergic neurons, ON‐ and OFF‐type cholinergic amacrine cells, exhibited different patterns of immunoreactivity for P2X_2_ receptors and CHT1 [27, 30]. CHT1 is expressed at a high level in ON‐type cholinergic amacrine cells and at a low level in OFF‐type cholinergic amacrine cells. On the other hand, P2X_2_ receptors are expressed at a low level in ON‐type cholinergic amacrine cells and at a high level in OFF‐type cholinergic amacrine cells. The present results strongly support the hypothesis that the main choline supply pathway for ACh synthesis differs between ON‐ and OFF‐type cholinergic amacrine cells.

In the retina, ATP is released from Müller glial cells [41, 42, 43], pigment epithelial cells [44], dopaminergic neurons [45], and cholinergic amacrine cells [46]. Among these neurons, reciprocal synapses between cholinergic amacrine cells are the likely release site of ATP because cholinergic amacrine cells also release ACh. Therefore, cholinergic amacrine cells may activate P2X_2_ receptors to recycle choline for ACh synthesis. Similarly, cholinergic neurons in the central and peripheral nervous systems have been shown to release ATP [25]. In addition, some cholinergic neurons express P2X_2_ receptors [47, 48, 49]. Therefore, the P2X_2_ receptor‐mediated ACh synthesis pathway may also be present in cholinergic neurons in other regions.

Based on our study series [27, 30], we suggest that choline entering the intracellular space through P2X_2_ receptors in cholinergic neurons is used to synthesize ACh. In the brain, disorders of the cholinergic system are considered to cause Alzheimer's disease, Parkinson's disease, and schizophrenia [50, 51, 52, 53, 54, 55]. Therefore, the present results may open new frontiers for the therapeutic manipulation of the cholinergic system in the brain.

## Conflict of interest

The authors declare no conflict of interest.

## Author contributions

MK, TM, TI, and YK designed the project. TM performed immunocytochemistry. TM prepared samples for the HPLC analysis. AM and YK performed the HPLC analysis. TM performed the western blot analysis. TM, TI, and MK wrote the manuscript. All authors have read and approved the final manuscript.

## Data Availability

The data are available from the corresponding author upon reasonable request.
